# Editorial of Special Issue “Molecular Mechanisms of Natural Products and Phytochemicals in Immune Cells and Asthma”

**DOI:** 10.3390/ijms24065913

**Published:** 2023-03-21

**Authors:** Young-Cheol Lee

**Affiliations:** Department of Herbology, College of Korean Medicine, Sangji University, 83 Sangjidae-gil, Wonju 26339, Gangwon-do, Republic of Korea; lyc072@sangji.ac.kr; Tel.: +82-33-730-0672; Fax: +82-33-730-0653

The Special Issue “Molecular Mechanisms of Natural Products and Phytochemicals in Immune Cells and Asthma” in the *International Journal of Molecular Sciences* includes original research and reviews on the molecular mechanisms of active, natural products (medicinal plants and animal ones) and phytochemicals in vitro and in vivo.

Allergic asthma, an increasingly common immunologic disease in industrialized countries, is a chronic inflammatory condition of the airways that causes airway hyperresponsiveness (AHR). A Th1-Th2 cytokine imbalance has been hypothesized to underlie allergic asthma through a change in immune responses from a Th1 profile to a Th2 (IL-4, IL-5, and IL-13) profile. Additionally, the recruitment of inflammatory cells such as T cells, mast cells, eosinophils, epithelial cells, macrophages and neutrophils is mediated via a number of chemokines and their receptors. Chemokines and their receptors are important therapeutic targets in asthma and allergic diseases because of their key role in immune cell recruitment and activation during inflammation [1]. In particular, Th2 type inflammation is the most important pathological pathway in asthma [2]. Many herbal extracts and their phytochemicals are effective medicines for asthma and respiratory diseases worldwide [3].

Two highly relevant papers have recently been published in the Special Issue of Bioactives and Nutraceuticals entitled “Molecular Mechanisms of Natural Products and Phytochemicals in Immune Cells and Asthma”. These two papers exhibit antiallergic and antiasthmatic effects of the active components in natural products.

The first article, “Antiallergic Effects of N,N’-dicoumaroylspermidine Isolated from *Lithospermum erythrorhizon* on Mast Cells and Ovalbumin-Induced Allergic Rhinitis” by Le et al. [4] revealed that a major compound of *Lithospermum erythrorhizon*, N,N’-dicoumaroylspermidine, inhibited the release of β-hexosaminidase, as well as the production of Th2 cytokines (IL-3, IL-4, and IL-13) by IgE-sensitized and BSA-stimulated RBL-2H3 cells. Using the allergic rhinitis mouse model, they showed that N,N’-dicoumaroylspermidine reduced the number of inflammatory cells in nasal lavage fluid and the production of serum OVA-specific IgE. N,N’-dicoumaroylspermidine isolated from *Lithospermum erythrorhizon* exhibits antiallergic properties, and it can be potentially effective for allergic rhinitis.

The second article, “Gypenoside A from *Gynostemma pentaphyllum* Attenuates Airway Inflammation and Th2 Cell Activities in a Murine Asthma Model” by Huang et al. [5] investigated the antiasthmatic effects of Gypenoside A, which is isolated from *G. pentaphyllum*. The study revealed that gypenoside A significantly reduced the expression of inflammatory genes and proteins in the lungs and bronchoalveolar lavage fluid and Th2 cytokine expression. Moreover, gypenoside A significantly inhibited the secretion of chemokines and inflammatory cytokines in tracheal epithelial cells. Gypenoside A is a natural phytochemical that can effectively attenuate airway hyperresponsiveness (AHR) and inflammation in asthma, mainly by blocking Th2 cytokines production. In summary, the two papers successfully demonstrate the antiallergic effects of phytochemicals from *Lithospermum erythrorhizon* roots and *Gynostemma pentaphyllum.*

Recently, natural products and their phytochemicals play increasingly critical roles, especially multi-ingredient synergistic effects [6]. The therapeutic efficacies of some natural products may arise from synergistic actions of their ingredients [7,8]. Therefore, this consideration should be taken into account in future.

We hope that this Special Issue can provide a comprehensive overview of the current state of research on molecular mechanisms of natural products and phytochemicals in immune cells and asthma.

In conclusion, the Guest Editor would like to thank all authors for their remarkable contributions and the Editorial Board for their support.
